# Therapeutic efficacy of a galactoglucan from *Pleurotus citrinopileatus* in constipation: modulation of aquaporin signaling and intestinal barrier

**DOI:** 10.3389/fnut.2025.1635487

**Published:** 2025-07-14

**Authors:** Yi Gao, Lan Deng, Yuanyuan Chen, Peiyou Qin, Yuanyuan Zhao, Xiaoyan Zhao, Wei Liu, Dan Wang, Shuang Zhao

**Affiliations:** ^1^Institute of Agri-food Processing and Nutrition, Beijing Academy of Agriculture and Forestry Sciences, Beijing, China; ^2^Department of Stomatology, Beijing Xicheng District Health Care Hospital for Mothers and Children, Beijing, China; ^3^College of Food Science and Bioengineering, Tianjin Agricultural University, Tianjin, China; ^4^College of Life Science and Technology, Mudanjiang Normal University, Mudanjiang, China; ^5^Institute of Plant Protection and Microbiology, Zhejiang Academy of Agricultural Sciences, Hangzhou, China

**Keywords:** aquaporin, constipation, *Pleurotus citrinopileatus*, polysaccharide, tight junction proteins

## Abstract

**Introduction:**

Constipation is a prevalent gastrointestinal disorder demanding effective therapeutic strategies. This study investigated the therapeutic potential of *Pleurotus citrinopileatus* polysaccharide (PCP-g), a novel galactoglucan, against sucralfate-induced constipation murine model, focusing on intestinal motility, fecal parameters, aquaporin signaling, and gut microbiota modulation.

**Methods:**

PCP-g was purified from *P. citrinopileatus* and its physicochemical properties were characterized. To evaluate the effects of PCP, the research utilized intestinal motility assays, fecal analysis, and *in vitro* fermentation. The role of Aquaporin 3 (AQP3) in constipation, especially regarding the PKA - phosphorylation mechanism, was investigated. The influence of PCP-g on PKA, phosphorylated PKA, AQP3, and tight junction proteins were examined at both the mRNA and protein levels.

**Results:**

PCP-g was identified as a homogeneous galactoglucan with a molecular weight of 7.49 × 10^3^ kDa, characterized by a backbone consisting of 1→4-linked glucose (Glcp) and branches of mannose (Manp) and Glcp. The composition of PCP-g includes Glc, Gal, Man, L-Fuc, Rha, GlcA, and Ara, in a molar ratio of 1.00:0.16:0.13:0.01:0.006:0.005:0.006. The oral administration of PCP-g resulted in a significant reduction in constipation symptoms, as indicated by an increase in fecal water content, normalization of pellet formation, enhancement of total fecal mass, decreased latency to the first stool, and improved intestinal propulsion. Furthermore, PCP-g was found to elevate the production of short-chain fatty acids (SCFAs) while simultaneously reducing intestinal gas. Mechanistically, PCP-g suppressed the PKA-dependent phosphorylation of AQP3, leading to the downregulation of AQP3 overexpression and enhanced colonic epithelial permeability. Concurrently, PCP-g reduced the expression of tight junction proteins ZO-1 and Occludin, contributing to the increase in fecal water content.

**Discussion:**

PCP-g effectively alleviates constipation by enhancing intestinal motility and fecal hydration. It modulates the PKA-AQP3 signaling pathway to improve colonic water permeability and positively influences the gut environment through the generation of SCFAs. These findings suggest that PCP-g may serve as a promising therapeutic candidate for the treatment of constipation, operating through aquaporin signaling and the regulation of the gut environment. The study advocates for further clinical trials and highlights the potential of edible mushroom polysaccharides in the management of constipation.

## 1 Introduction

Functional constipation is defined as constipation caused by slow colonic transit and intestinal dysfunction, without any underlying organic disease (1, 2). This condition may trigger a range of variety gastrointestinal issues, including stomach discomfort, reduced appetite, and sensations of nausea. Furthermore, it can contribute to mental health challenges such as anxiety and depression, which can subsequently affect the patient’s daily functioning (3). Although slow transit constipation (STC) is a prevalent condition worldwide, its underlying causes remain inadequately understood. Numerous studies have indicated a multitude of contributing factors to the onset of STC, including alterations in the intestinal nervous system and metabolic processes, abnormalities in mucosal secretions, disruptions in the balance of gut microbiota, and irregularities in gastrointestinal hormone levels, among others (4, 5). Constipation is often regarded as an indicator of an underlying medical condition rather than an independent disease. Therapeutic approaches may encompass dietary modifications, the utilization of laxatives, the implementation of enemas, the application of biofeedback techniques, and, in certain instances, the exploration of surgical interventions (6). Nevertheless, these treatment modalities often demonstrate limited efficacy and may be associated with dependency and adverse effects, thereby presenting a significant challenge in clinical management.

The transit of water within the digestive system is crucial for sustaining water homeostasis and regulating fluid and electrolyte equilibrium, and it also influences the occurrence of constipation (7). The colon is responsible for the absorption of water and electrolytes, the formation of feces, and the secretion of an adequate amount of mucus to facilitate the smooth passage of feces (8). However, excessive absorption of water from the stool by the colon can result in the desiccation and hardening of stool, ultimately leading to constipation. Aquaporins (AQPs) located in the tissues of the digestive system, which is regulated by cAMP/PKA, play a significant role in water metabolism and homeostasis (9). Meanwhile, tight junction proteins (TJPs) in the intestinal tract are crucial for regulating water absorption and secretion, maintaining electrolyte and water balance, and influencing water metabolism (10), all of which can impact the development of constipation.

Intensive studies have demonstrated that polysaccharides derived from edible fungi and their complexes are characterized by immunoregulatory properties, minimal side effects, and multiple therapeutic targets (11–14). These compounds highlight the modulation of gut microbiota and the enhancement of intestinal function in the treatment of constipation (15–18). *Pleurotus citrinopileatus*, a valuable edible fungus known for its delicious taste and nutritional benefits in the Eastern world, contains several primary active components, notably polysaccharides and polysaccharide complexes. These components exhibit a range of biological activities, including hypoglycemic effects (19), anti-inflammatory properties (20), anti-tumor activity (21), and regulation of gut microbiota (22), in addition to their immunomodulatory effects (23, 24). Our previous research has demonstrated that the polysaccharide peptides PSI and PSII extracted from *P. citrinopileatus* are effective in modulating gut microbiota *in vitro* (15). Therefore, it is speculated that polysaccharides from *P. citrinopileatus* may serve as potential therapeutic agents for constipation.

Although the efficacy of polysaccharides derived from *P. citrinopileatus* (PCP) in enhancing intestinal function has been established, there remains a paucity of information regarding its potential to alleviate constipation and the mechanisms underlying its effects. This study involved the isolation and characterization of a purified galactoglucan (PCP-g) to assess its physicochemical properties. We investigated the laxative effects and mechanisms of PCP-g in a murine model of sucralfate-induced constipation by analyzing the expression of key factors within the cAMP/PKA/AQP3 signaling pathway and TJPs. Our results demonstrated that PCP-g promoted intestinal peristalsis, improved defecation patterns, diminishes gas production within the intestines, and alleviates bloating. Furthermore, it regulated the expression of aquaporin proteins and tight junction proteins, which contributed to an increase in fecal and intestinal water content, thereby alleviating constipation. This research provided the groundwork for the potential clinical application of PCP-g in laxative therapies.

## 2 Materials and methods

### 2.1 Chemicals and reagents

*Pleurotus citrinopileatus* fruiting bodies were preserved at Beijing Academy of Agricultural and Forestry Sciences (Beijing, China). DE-52 was purchased from Solarbio (Beijing, China), and the Superdex-75 column was acquired from the General Electric Company (GE, USA). Sulphoaluminate sugar and was purchased from Shanghai Huayuan Anhui Renji Pharmaceutical Co., Ltd. Fructooligosaccharide was purchased from Shanghai Jijing Biotechnology Co., Ltd. The BCA protein assay kit, Western blot kit, primers, and all other reagents used in RT-PCR were purchased from Sangon Biotech (Shanghai, China). AQP3 and Occludin antibodies were obtained from Abcam (Cambridge, UK), while PKA, β-actin, and phospho-PKA antibodies were obtained from Cell Signaling Technology (Danvers, MA, USA). All other chemicals and solvents used were of analytical reagent grade and obtained from Sinopharm Chemical Reagent Co., Ltd. (Shanghai, China).

### 2.2 Extraction and purification of PCP-g

The crude polysaccharide, PCP-g, was extracted by water extraction and alcohol precipitation method, consistent with previously reported procedure (25). The crude polysaccharide was dissolved in distilled water and applied to a DE-52 column (1 cm × 30 cm) equilibrated with a 10 mM NH_4_HCO_3_-NH_3_H_2_O buffer solution (pH 9.4). The column was eluted sequentially with 0 and 0.2 M NaCl solutions at a flow rate of 2 mL/min. The polysaccharide concentration of each elution peak was determined using the sulfuric acid-phenol method, and the elution peaks were defined as D1 and D2. The unadsorbed peak D1, which contained a high concentration of polysaccharide, was further processed using a Superdex-75 (16/60) column equilibrated with ultrapure water, utilizing an AKTA Purifier (GE Healthcare). The first peak, enriched in polysaccharide, was collected, concentrated, and freeze-dried.

### 2.3 Physicochemical properties and structural characterization of PCP-g

#### 2.3.1 Monosaccharide composition and molecular weight analysis of PCP-g

The monosaccharide composition of PCP-g was examined using gas chromatography-mass spectrometry (GC-MS) as reported by Zhao et al. (25). The molecular weight and homogeneity of PCP-g was evaluated through high-performance gel permeation chromatography (GPC) employing TSK GMPWXL columns. Additionally, freeze-dried polysaccharide powder was analyzed at the Science Spectrum R&D Center located in Shandong, China.

#### 2.3.2 Methylation analysis of PCP-g

The methylation of PCP-g was modified in accordance with a previous published study (26, 27). Briefly, 10 mg of PCP-g was dissolved in high-purity water and subjected to a reaction with 0.2 M MES and 100 mg/mL carbodiimide. Following a 2-h incubation period, the reaction mixture was combined with 2 M imidazole and 30 mg/mL NaBD_4_ for 3 h. The reaction was subsequently terminated by the addition of acetic acid. After the glucuronic acid reduction reaction, the sample was dialyzed and freeze-dried, then dissolved in dimethyl sulfoxide (DMSO) and incubated with 1 mg of NaOH for 30 min, followed by a 1-h reaction with iodomethane. The methylated PCP-g was treated with 2M of trifluoroacetic acid at 121°C for 90 min and reduced using a mixture of 2 M ammonia and 1 M NaBD4. After 2.5 h, the reaction was terminated with acetic acid and the product was washed with methanol and dried under nitrogen. Subsequently, a 2.5-h reaction at 100°C with acetic anhydride, the reduction product of PCP-g was extracted using dichloromethane and analyzed by GC–MS on an Agilent 7890 B-7000D. The glucosidic patterns of the various products were determined by comparing the results with the GC/MS patterns from the Complex Carbohydrate Structure Database, which was established by the Complex Carbohydrate Research Center at the University of Georgia in the U.S.A.

### 2.4 Laxative effects of PCP-g on sucralfate-induced constipation

#### 2.4.1 Animals and treatments

Male C57BL/6 mice, aged 7–8 weeks, were obtained from Beijing Vital River Laboratory Animal Technology Co., Ltd. The animal experiments in this study were conducted in compliance with approved protocols and guidelines from the Institutional Animal Care and Use Committee of Beijing Life Biosciences Co., LTD (Number: SKLF-DWLL-20221213-01).

The mice were randomly divided into seven groups, consisting of ten mice. The groups included a negative control group, a fructooligosaccharide (FO) positive control group (1.0 g/kg), a model group, a low- dose PCP-g group (LP) (1.0 g/kg), and a high-dose PCP-g group (HP) (2.0 g/kg). The mice were acclimated for 1 week at a temperature of 22°C and a humidity level of 50%–60%. Starting on the eighth day, the low- and high-dose groups were administered a suspension of PCP-g via gavage daily. The LP group received a dosage of 1.0 g/kg per day, while the HP group was given 2.0 g/kg per day, as determined by the pre-experimental protocol. The FO positive control group was provided with a suspension of fructooligosaccharides at a dosage of 1.0 g/kg. Both the negative control and model groups received an equivalent volume of distilled water. This feeding protocol was maintained for a duration of 14 days. Throughout the experiment, the mice were fed normally, and their morphology and respiratory characteristics were observed daily. Following the final gavage, the mice in each group were fasted for 16 h with water and housed individually. The mice in the model, FO, and PCP-g groups received a gavage of 5 mg/kg of sucralfate suspension, while the negative control group was administered the same volume of distilled water via gavage.

#### 2.4.2 Gastrointestinal motility test

Gastric motility tests were conducted utilizing the following methodology. Thirty minutes subsequent to the establishment of sucralfate-induced constipation model in the mice, both the negative control and model groups were administered an identical dose of carmine solution. Concurrently, the positive control group and the treatment group received the corresponding drug carmine solution. Following a 25-min period of intragastric administration, the mice from each group were sacrificed by cervical dislocation. The abdominal cavity was then opened, and the intestine was excised from the pylorus to the cecum without any traction. The total length of the small intestine was measured after it was laid flat. The distance covered by charcoal in the small intestine from the pylorus to the cecum was measured, and the ratio of distance traveled by the carmine to the length of the small intestine was calculated using the following formula.


$$\mathrm{GI}\mathrm{transit}\mathrm{ratio}\left(\%\right)=\frac{\mathrm{Distance}\,\mathrm{traveled}\,\mathrm{by}\,\mathrm{the}\,\mathrm{carmine}}{\mathrm{Total}\,\mathrm{length}\,\mathrm{of}\,\mathrm{small}\,\mathrm{intestine}\,\left(\mathrm{cm}\right)}\times 100\%$$

#### 2.4.3 Laxative activity test

The remaining five mice in each group were used for the small intestine feces experiment. After 30 min of establishing the constipation model in the mice, the carmine solution corresponding to the drugs in the FO positive control and treatment groups was administered, while the negative control and model groups received only the same dose of carmine solution. Following a normal diet, the time at which red feces first appeared after administration was continuously observed for each animal, and the number of red feces produced by each mouse within 5 h was recorded. The total weight of red feces (W1) for each mouse was measured, and W2 was determined after drying. The water content of the feces produced within 5 h by each mouse was calculated using the following formula.


Fecalwatercontent(%)=

$$\frac{\left(\mathrm{Weight}\,\mathrm{of}\,\mathrm{wet}\,\mathrm{feces}-\mathrm{Weight}\,\mathrm{of}\,\mathrm{dry}\,\mathrm{feces}\right)}{\mathrm{Weight}\,\mathrm{of}\,\mathrm{wet}\,\mathrm{feces}}\times 100\%$$

### 2.5 Gut microbiota metabolism analysis

The feces from each group of mice were collected, and a fecal suspension with a mass concentration of 100 g/L was prepared using PBS buffer. This suspension was then filtered through a 0.125 mm sterile metal sieve to remove large food residues, resulting in the preparation of the basic medium (YCFA). The fecal suspension was inoculated into the basic medium and cultured in a constant temperature incubator at 37°C for 24 h.

#### 2.5.1 Microbiota gas production analysis

The gas samples produced by *in vitro* batch culture fermentation were collected after 24 h of fermentation. The volumes of methane (CH_4_), ammonia (NH_3_), hydrogen sulfide (H_2_S), hydrogen (H_2_), and carbon dioxide (CO_2_) in the medium were measured using a gas analyzer.

#### 2.5.2 SCFAs quantification

The fermentation broth was centrifuged at 9000 r/min for 3 min. After centrifugation, the supernatant was used for the analysis of SCFAs metabolism. The SCFAs were identified in the supernatant using GC with a Shimadzu GC-2010 Plus (Japan). The GC analyses were performed using a DB-FFAP column (Agilent Technologies, USA) in conjunction with a hydrogen flame ionization detector. Acetic, propionic, isobutyric, butyric, pentanoic, isopentanoic, and caproic acids were obtained from Sigma.

### 2.6 Histopathological examination

The intestinal tissue underwent fixation with formaldehyde, followed by trimming, dehydration, and embedding in paraffin. Subsequently, it was sectioned into slices approximately 10 μm thick. These sections were then subjected to dewaxing, stained with hematoxylin and eosin (H&E), and analyzed using an optical microscope (ECLIPSE CI-L; Nikon, Tokyo, Japan).

### 2.7 Real-time RT-PCR

Total RNA was isolated from the murine colonic tissue using Trizol reagent following the manufacturer’s instructions. Reverse transcription was conducted using Moloney murine leukemia virus reverse transcriptase for complementary DNA synthesis. Real-time PCR reactions were carried out using a SYBR Premix Ex Taq Kit in an ABI 7500 system. The primer sequences are presented in Table 1, which included PKA, AQP3, Occludin, and ZO-1. The relative expression levels of the genes were determined using the 2-ΔΔCT method (28), employing β-actin as the reference gene.

**TABLE 1:** Primer sequence.

Genes	Forward	Reverse
PKA	5′-ACATCCAGGTCACAGATT-3′	5′-CCACCGCCTTATTGTAAC-3′
AQP3	5′-GCTATGACCCTTGACTGGGG-3′	5′-AGTGGAGTTTCCCACCCCTA-3′
Occludin	5′-ATCCTGTCTATGCTCATTATTG-3′	5′-GGTCTGTATATCCGCCATA-3′
ZO-1	5′-CATCATTCGCCTTCATACAA-3′	5′-ACACAACCTCATCCTCATT-3′
β-actin	5′-CTGTGCCCATCTACGAGGGCTAT-3′	5′-TTTGATGTCACGCACGATTTCC-3′

### 2.8 Western blot

Tissue was homogenized in a lysis buffer containing 50 mM Tris-HCl (pH 7.4), 1 mM EDTA, 150 mM NaCl, 1% Triton X-100, 1% sodium deoxycholate, 0.1% SDS, 1 mM trichostatin A, and a phosphatase inhibitor cocktail. The protein concentration was determined using a BCA protein quantification kit. Proteins were separated by SDS-PAGE and subsequently transferred to a PVDF membrane. After blocking with 3% BSA/TBST at room temperature for 1 h, the membranes were incubated overnight at 4°C with primary antibodies specific for AQP3 (1:2,000), PKA (1:2,000), PKA C-α (1:1,000), occludin (1:1,000), and β-actin (1:2,000). Following three washes with TBST, the membranes were incubated with an HRP-labeled secondary antibody at a dilution of 1:2,000. The blots were washed three additional with TBST buffer, and the immunoreactive bands were detected using an enhanced chemiluminescence method. The results were normalized to β-actin expression, which served as the internal standard (29).

### 2.9 Statistical analysis

All data are expressed as mean ± SEM. The data were analyzed using a one-way ANOVA, followed by *post hoc* Dunnett’s or Turkey’s test. Data with *P* < 0.05 was believed to possess significant difference. The grayscale targets of the standard protein bands were analyzed using Image J.

## 3 Results

### 3.1 Purification and structural analysis of PCP-g

**FIGURE 1:**
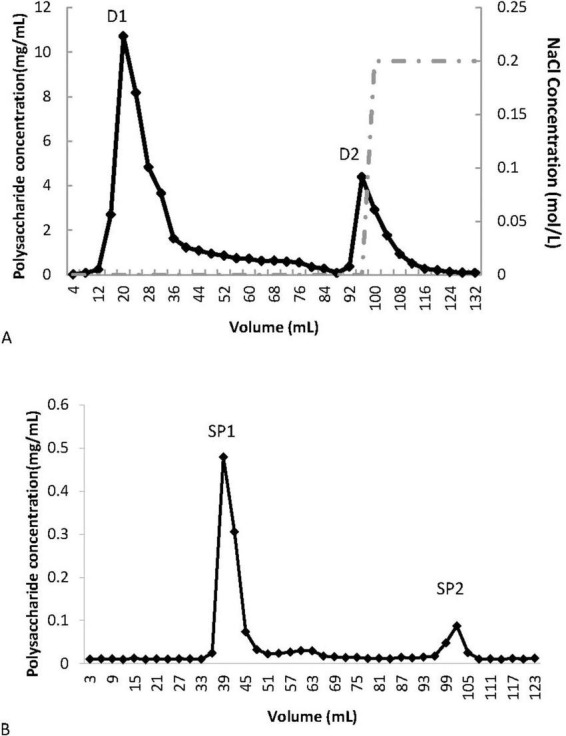
Purification of PCP-g from *P. citrinopileatus*. **(A)** Crude polysaccharide separation via DE-52 cellulose column chromatography. **(B)** Further purification of fraction D1 using Sephadex G-75 gel filtration.

The crude polysaccharide from *P. citrinopileatus* was obtained through a process of water extraction followed by alcohol precipitation from the fruiting bodies. After the removal of free proteins, a DE-52 column was employed to separate the components, with D1 and D2 collected via elution using 0 and 0.2 M NaCl in an NH_4_HCO_3_-NH_3_H_2_O buffer (10 mM, pH 9.4), respectively (Figure 1A). D1 was further purified using a Superdex-75 column to obtain SP1 and SP2 (Figure 1B). SP1, which had the highest yield, was designated as PCP-g, with an extraction rate of approximately 1.2%.

### 3.2 Physicochemical properties and structural characterization of PCP-g

The molecular weight of PCP-g was assessed by high-performance gel permeation chromatography (HPGPC) analysis. The molecular weight parameters, including the number-average molecular weight (Mn), weight-average molecular weight (Mw), and peak molecular weight (Mp) were presented in Table 2. The PCP-g exhibited a single peak, with a number average molecular weight of 6.17 × 10^3^ kDa and a weight average molecular weight of 7.49 × 10^3^ kDa. Furthermore, the GC chromatogram of the standard monosaccharide mixture and the composition of PCP-g were presented in Supplementary Figure 1 and Table 3. The results indicated that PCP-g was primarily composed of glucose, which constituted 96.8% of its total composition. Additionally, it also contained trace amounts of galactose, mannose, fucose, rhamnose, glucuronic acid, and arabinose, with a molar ratio of 1.00: 0.16: 0.13: 0.01: 0.006: 0.005: 0.006.

**TABLE 2:** The molecular weight of PCP-g.

PCP-g	Molecular weight parameters (Da)	Mw/Mn
Mp	7772833	1.21
Mn	6173761	
Mw	7498604	
Mz	8522992	

**TABLE 3:** Monosaccharide composition of PCP-g.

Monosaccharide names	Content ratio (%)	Molar ratio
Mannose	1.30	0.13
Rhamnose	0.06	0.006
Glucuronic acid	0.05	0.005
Glucose	96.80	1.00
Galactose	1.66	0.16
Arabinose	0.05	0.006
Fucose	0.09	0.01

As shown in Supplementary Figure 2, 11 sugar residues were identified in total ion chromatography (TIC) by methylation analysis of PCP-g, which have been summarized in Table 4. The methylation analysis of PCP-g identified 11 distinct glycosidic linkages via total ion chromatography (TIC), revealing a structurally complex β-glucan framework. The predominant motif, →4)-Glcp-(1→(64.38%), established a backbone dominated by β-(1→4)-linked glucose residues, consistent with the monosaccharide composition (96.8% glucose, Table 3). A significant proportion of →4,6)-Glcp-(1→(10.35%) and terminal Glcp (16.53%) indicated a branched architecture, where glucose units form both the linear backbone and non-reducing termini. Minor linkages, including →6)-Galp-(1→(2.65%) and →6)-Manp-(1→(0.68%), suggested galactose and mannose side chains attached via 1,6-linkages. Complex branching was further supported by →3,4)-Glcp-(1→(2.10%) and →3,4,6)-Glcp-(1→(0.69%), pointing to trifunctional glucose residues at branching nodes. Terminal residues (T-Manp: 1.08%; T-Glcp: 16.53%) likely marked chain termini or branch endpoints. Collectively, PCP-g was characterized as a highly substituted β-(1→4)-glucan with a linear backbone periodically interrupted by 1,6-linked galactose/mannose side chains and sporadic branching at positions 3,4 or 3,4,6 of glucose. The dominance of glucose in both backbone and termini aligns with its monosaccharide profile, while trace galactose (1.66%) and mannose (1.30%) reflected their roles as minor substituents.

**TABLE 4:** Methylation analysis of PCP-g.

Linkage types	Methylate alditol acetate	Retention time (min)	Ratio (%)
T-Manp	1,5-Di-O-acetyl-1-deuterio-2,3,4,6-tetra-O-methyl-D-mannitol	18.5486	1.076
T-Glcp	1,5-Di-O-acetyl-1-deuterio-2,3,4,6-tetra-O-methyl-D-mannitol	18.6815	16.527
3-Glcp	1,3,5-Tri-O-acetyl-1-deuterio-2,4,6-tri-O-methyl-D-glucitol	22.5931	0.182
2-Glcp	1,2,5-Tri-O-acetyl-1-deuterio-3,4,6-tri-O-methyl-D-galactitol	22.8145	0.749
6-Manp	1,5,6-Tri-O-acetyl-1-deuterio-2,3,4-tri-O-methyl-D-mannitol	24.2611	0.678
4-Glcp	1,4,5-Tri-O-acetyl-1-deuterio-2,3,6-tri-O-methyl-D-mannitol	24.7186	64.379
6-Galp	1,5,6-Tri-O-acetyl-1-deuterio-2,3,4-tri-O-methyl-D-galactitol	26.0722	2.652
3,4-Glcp	1,3,4,5-Tetra-O-acetyl-1-deuterio-2,6-di-O-methyl-D-glucitol	26.8639	2.095
4,6-Glcp	1,4,5,6-Tetra-O-acetyl-1-deuterio-2,3-di-O-methyl-D-galactitol	28.9896	10.345
2,5-Araf	1,2,4,5-Tetra-O-acetyl-1-deuterio-3-O-methyl-D-arabinitol	30.2492	0.623
3,4,6-Glcp	1,3,4,5,6-Penta-O-acetyl-1-deuterio-2-O-methyl-D-mannitol	31.3367	0.694

The ratios were calculated based on the peak areas.

### 3.3 Laxative effects of PCP-g intervention

**FIGURE 2:**
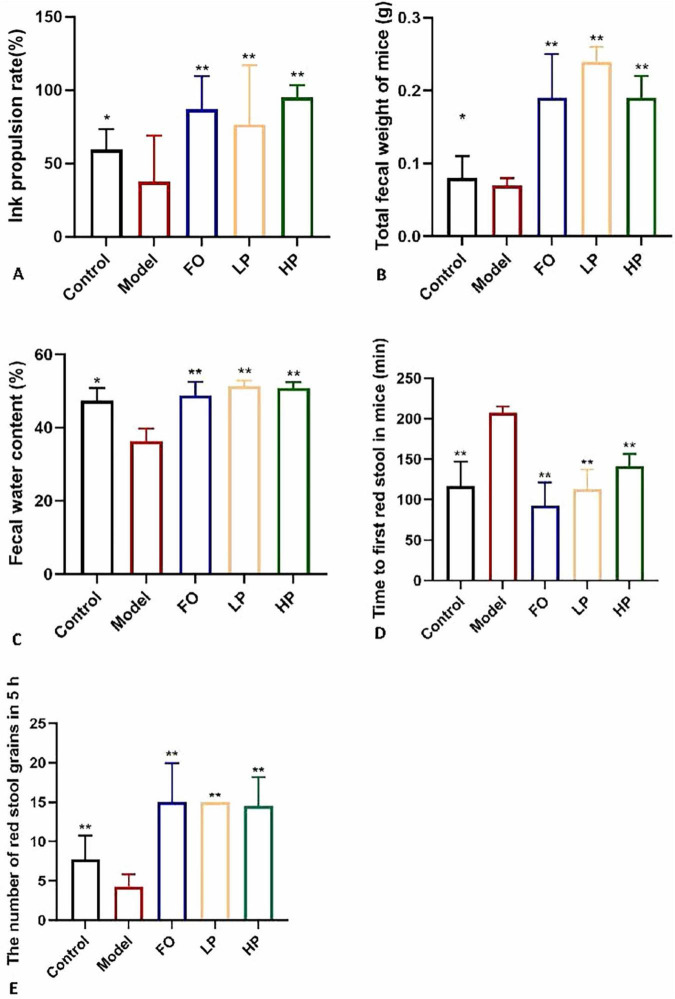
Laxative effects of PCP-g intervention. **(A)** Intestinal peristalsis rate. **(B)** Fecal output. **(C)** Fecal moisture content. **(D)** Initial defecation latency. **(E)** Number of red-stained stools. Data are presented as mean ± SD; *P < 0.05 and **P < 0.01 vs. model group.

The intestinal peristalsis was accelerated by PCP-g intervention, as shown in Figure 2A. Following the administration of sucralfate to induce constipation in mice, the small intestine carmine propulsion rate in the model group was significantly reduced by 36.6% compared to the negative control group (*P* < 0.05). This statistically significant difference indicated that sucralfate effectively inhibited small intestine peristalsis. Compared to the model group, the LP group showed a 102.9% improvement in propulsion rate (*P* < 0.01), while the HP group demonstrated the most pronounced therapeutic effect with a 152.1% increase (*P* < 0.01), which was greater than the FO positive control group, which showed an increase of 129.9%. These results demonstrated that PCP-g effectively ameliorates constipation-induced intestinal dysmotility, with maximal therapeutic efficacy observed at higher dosages.

As illustrated in Figures 2B–E, sucralfate-induced constipation modeling significantly reduced total fecal output (0.05 g decrease, *P* < 0.05), fecal water content (23.6% decrease vs negative control, 25.8% vs FO group; *P* < 0.01) and 5-h fecal pellet count (4.3 vs 7.7 in negative control, *P* < 0.01), accompanied by prolonged first defecation latency (207.7 min vs 117 min in negative control, *P* < 0.01) compared to the negative control, which confirmed successful model establishment. Both low- and high-dose PCP-g interventions demonstrated significant therapeutic effects. Compared to the model group, LP and HP groups increased total fecal mass by 0.17 g and 0.12 g (*P* < 0.01), elevated fecal water content by 41.9% and 40.6% (*P* < 0.01), and reduced first defecation latency to 112.5 min and 141.3 min (*P* < 0.01), respectively. The administration of PCP-g treatment resulted in an increase in fecal pellet counts, with HP group exhibiting an average of 14.5 pellets and LP group displaying an average of 15 pellets (*P* < 0.01). The FO group exhibited intermediate improvements across all parameters, underscoring PCP-g enhanced bioactivity. These results collectively demonstrated that PCP-g effectively alleviates constipation through dual mechanisms of promoting intestinal motility and maintaining fecal hydration, with high-dose administration showing particular advantage in enhancing colonic propulsion capacity.

### 3.4 PCP-g decreased the formation of fermentation gas

**FIGURE 3:**
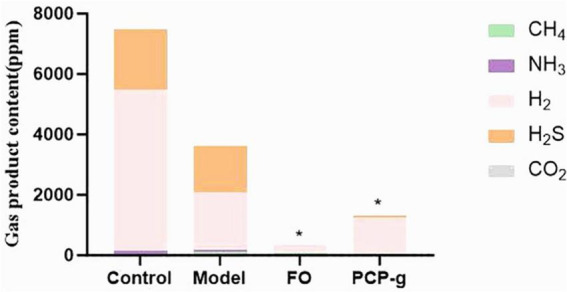
Total gas production during *in vitro* fermentation. *P < 0.05 and **P < 0.01 vs. model group.

The gas composition profiles generated during *in vitro* batch culture fermentation were quantitatively analyzed following a 24-h incubation period. The total gas production profiles across experimental groups were summarized in Figure 3. The results showed no statistically significant difference (*P* > 0.05) in total gas output between the negative control group and the model group. The differential effects were observed in the PCP-g group and FO group. Compared to the model group, hydrogen sulfide (H2S) levels were reduced by 99.67% (*P* = 0.001) and 97.3% (*P* = 0.001) for PCP-g and FO treatments, respectively. Similarly, ammonia (NH3) production decreased by 85% (*P* = 0.0015) and 90% (*P* = 0.0016), while methane (CH_4_) emissions declined by 34.6% (*P* < 0.0001) and 73.1% (*P* = 0.0292) for the respective interventions. In contrast, hydrogen (H_2_) and carbon dioxide (CO_2_) production remained unaffected across all groups (*P* > 0.05). These findings demonstrated that PCP-g effectively suppressed gas production by gut microbiota, thereby mitigating gastrointestinal flatulence.

### 3.5 PCP-g promoted the productions of SCFAs

**FIGURE 4:**
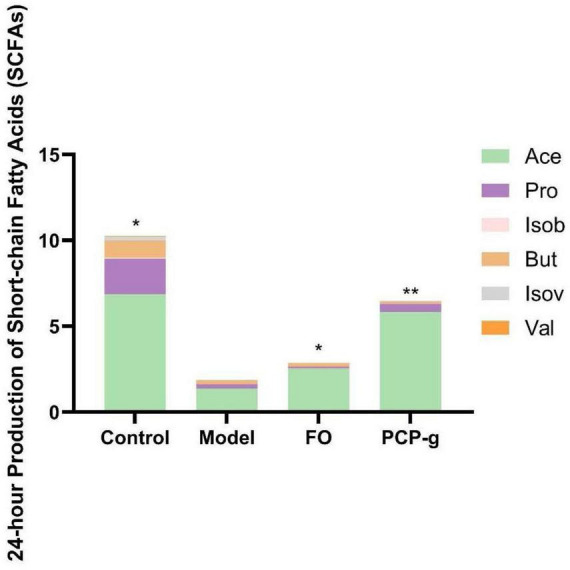
Short-chain fatty acids (SCFAs) production during *in vitro* fermentation. *P < 0.05 and **P < 0.01 vs. model group.

Following 24 h of *in vitro* fermentation, gas chromatographic analysis was performed to quantify SCFAs production. As shown in Figure 4, there was a significant reduction in the yields of acetic acid, propionic acid, and butyric acid in the model control group compared to the control group. It suggested that the induction of the constipation model substantially suppressed the synthesis of SCFAs. The therapeutic supplementation with PCP-g and FO elicited a pronounced elevation in acetic acid production, demonstrating 327.4% and 80.1% increase compared to the model group (*P* < 0.05). It implied that PCP-g modulated SCFAs metabolism in a constipation-specific context, particularly favoring acetic acid biosynthesis.

### 3.6 Histopathological analysis

**FIGURE 5:**
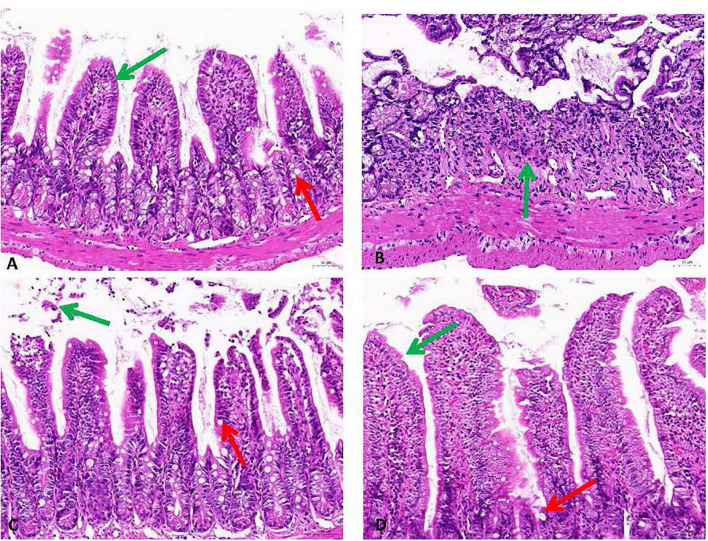
Histopathological analysis of intestinal tissues stained with H&E (200× magnification). The green arrows indicate the presence of mucosal epithelial cells, while the red arrows show cup cells. **(A)** Control group. **(B)** Model group. **(C)** FO group. **(D)** PCP-g group.

The histopathological evaluation of intestinal tissues via HE staining revealed constipation induced pathologic changes in intestinal outcome intestinal structure (Figure 5). Compared with the negative control group, the intestinal tissues of the model group showed disturbed arrangement of intestinal villi, large ulcerative necrosis was seen in the mucosal layer of the tissues, and the tissues were infiltrated with obvious inflammatory cells. In the PCP-g group, intestinal villi exhibited well-organized alignment with preserved mucosal architecture, showing no evidence of epithelial shedding, necrosis, or submucosal edema. Additionally, inflammatory cell infiltration was absent in the tissue, indicating structural and functional integrity of the intestinal mucosa. The therapeutic efficacy of PCP-g in preserving mucosal integrity and mitigating pathological alterations significantly surpassed that of the FO group.

### 3.7 PCP-g modulated the PKA/AQP3 signaling axis and tight junction proteins dynamics

**FIGURE 6:**
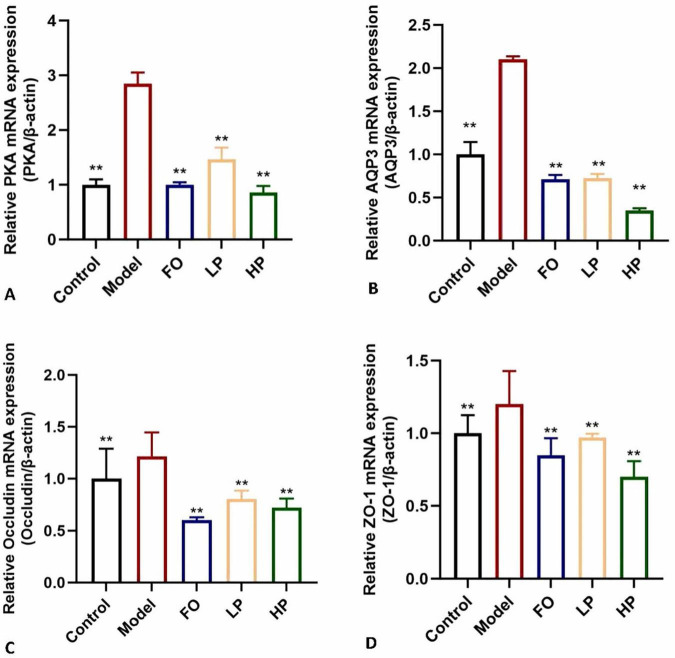
Modulation of mRNA expression by PCP-g. **(A)** Protein Kinase A (PKA). **(B)** Aquaporin-3 (AQP3). **(C)** Occludin. **(D)** Zonula Occludens-1 (ZO-1). *P < 0.05 and **P < 0.01 vs. model group.

The expressions of several key regulators associated with colonic water absorption were determined. As illustrated in Figure 6, the mRNA expression levels of the four target genes were significantly up-regulated in the model group compared to the negative control group (*P* < 0.05). This coordinated overexpression pattern suggested compensatory mucosal adaptation to chronic dehydration stress. PCP-g intervention effectively reversed these pathological alterations. In contrast, the expression levels of PKA, AQP3, ZO-1, and Occludin were significantly decreased in the LP group compared to the model group (*P* < 0.05). Notably, the contents of PKA, AQP3, and ZO-1 in the HP group were significantly lower than those in the FO group (*P* < 0.05). Moreover, the expression of Occludin in the HP group was significantly lower than that in the FO group, with extremely significant differences (*P* < 0.01). The differential expression profiles (Figure 6) established that PCP-g modulated fluid homeostasis through the PKA/AQP3 axis and epithelial barrier function via ZO-1/Occludin coordination pathways to alleviate constipation. These findings suggested that PCP-g exhibited a superior therapeutic effect compared to fructooligosaccharides.

**FIGURE 7:**
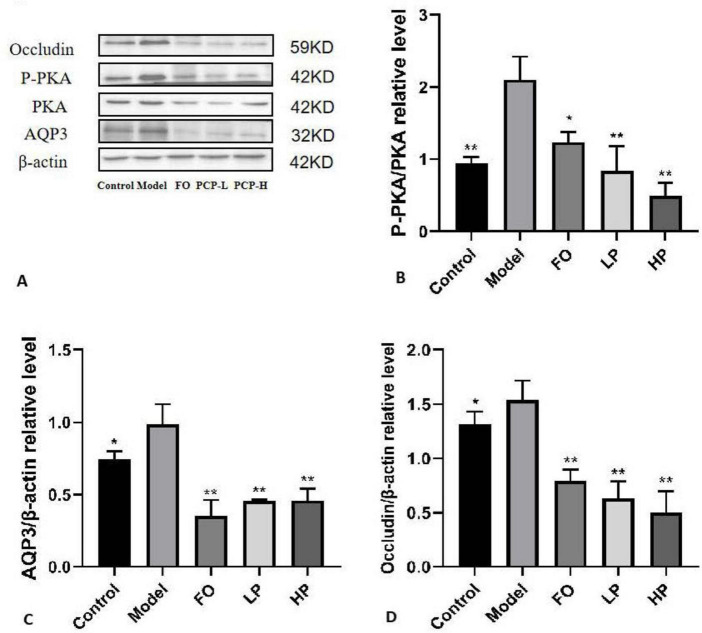
Western blot analysis of colon protein levels. **(A)** Representative blot images. **(B)** Relative expression of phosphorylated PKA (P-PKA). **(C)** Relative expression of AQP3. **(D)** Relative expression of Occludin. *P < 0.05 and **P < 0.01 vs. model group.

Based on the transcriptional findings, complementary protein-level evidence was obtained through immunoblotting analysis (Figure 7). The model group exhibited pathologically elevated phosphorylated PKA (p-PKA), AQP3, and Occludin levels relative to the negative control group (*p* < 0.05), reinforcing the activation of colonic dehydration pathways. The intervention of PCP-g dose-dependently attenuated these elevations, with high-dose PCP-g demonstrating superior suppression of p-PKA compared to FO treatment (*p* < 0.05). Notably, while fructooligosaccharides and PCP-g equivalently downregulated AQP3 expression (*p* < 0.01), only PCP-g exerted graded modulation of Occludin restoration contingent on polysaccharide concentration. The biphasic regulation, which simultaneously inhibited PKA/AQP3-mediated hyperabsorption and reconstituted Occludin-dependent barrier integrity, provided validation of PCP-g’s dual-targeting efficacy in constipation mitigation, thereby corroborating the mRNA-level observations from Figure 6.

## 4 Discussion

Functional constipation, a multifactorial gastrointestinal disorder, arises from genetic predispositions, neuropsychiatric influences, and environmental factors including dietary habits, intestinal dysbiosis, and impaired colonic motility (30, 31). *P. citrinopileatus* is widely consumed as a food and medical edible mushroom in China and eastern Asia, which is particularly rich in dietary fibers that exhibit prebiotic properties capable of modulating gut microbiota composition, enhancing intestinal motility, and demonstrating therapeutic potential for constipation relief.

In this study, the laxative polysaccharide PCP-g, which has a molecular weight of 7.49 × 10^6^ Da, was isolated and purified from the fruiting bodies of *P. citrinopileatus*. In the laxative experiments, the results indicated that PCP-g could alleviate sucralfate-induced constipation in mice, with the significant increase of GI transit rate, the fecal number, and fecal water content, as well as shortening the time to first red stool. By contrast, PCP-g had a better alleviating effect than positive control FO. PCP-g, as a water-soluble polysaccharide, consistent with previously reported water-soluble dietary fibers, improved bowel habits, softened stools, and increased the frequency of bowel movements (32–34).

The methylation analysis of PCP-g revealed its unique and complex β-glucan framework, providing significant insights into the structure-function relationship of polysaccharides. The predominant →4)-Glcp-(1→ glycosidic bonds (64.38%) formed the core backbone, which resembles the backbone of cellulose, suggesting that PCP-g may exhibit certain physical stability. From a biological perspective, the abundant β-(1→4) glycosidic linkages may influence the rate and mode of degradation by intestinal microbiota (35), thereby modulating prebiotic activity (36). Branching structures in polysaccharides are often associated with various biological activities, such as enhanced antioxidant (37) and immunomodulatory effects (36). The presence of branching points increases the surface area of PCP-g, enhancing its binding ability to receptors or altering its conformation in solution. This, in turn, influences interactions with intestinal epithelial cells (38). The identification of the →3,4)-Glcp-(1→ and →3,4,6)-Glcp-(1→ glycosidic bonds further confirms the complexity of the branching structure of PCP-g. Such intricate branching patterns are uncommon in other bioactive polysaccharides and may endow PCP-g with unique biological activities. For instance, similar complex branching structures in *Ganoderma* polysaccharides have been demonstrated to be closely associated with antitumor activity (39–41). Therefore, the branching structure of PCP-g may serve as a crucial structural basis for its potential physiological functions, such as the treatment of constipation. The methylation analysis combined with the GC-MS technique employed in this study provided a powerful tool for revealing the structural properties of PCP-g. However, it lacks information regarding the spatial conformation of PCP-g, which also influences its biological activity and therapeutic effects on constipation. Future studies could investigate the relationship between these branching structures and aquaporin signaling regulation, as well as the association between spatial conformation and therapeutic efficacy.

During anaerobic fermentation in the gut, the microbiota produces SCFAs such as butyrate, acetate, and propionate. Acetic acid plays an important role in the absorption of water and electrolytes in the intestine, which relieves constipation by increasing acetic acid-producing bacteria that were correlated positively with the small intestinal transit rate and water content of feces (42). Butyrate also can increase butyric acid-producing bacteria that were correlated negatively with the time to the first colored stool defecation (43). SCFAs can regulate the structure of the gut microbiota, affect water absorption, alter the gas production capacity of intestinal microorganisms, and thereby alleviate constipation (44). The findings of our study indicated that therapeutic supplementation with PCP-g resulted in a significant increase in acetic acid production, demonstrating a 327.4% enhancement relative to the model group (*P* < 0.05). It suggested that PCP-g might ameliorate constipation through the modulation of water metabolism.

Recent findings identify two primary mechanisms for the absorption of water in the gastrointestinal tract: paracellular transport, which occurs through intercellular tight junctions, and transcellular transport, which involves the movement of water across epithelial membranes (45, 46). In the paracellular transport pathway, tight junction integrity serves as a bidirectional modulator of intestinal water flux. TJPs such as ZO-1 and Occludin play a significant role in the regulation of intestinal water transport. When the integrity of tight junctions is compromised, resulting in the formation of gaps, there is an associated increase in the permeability of epithelial cells (47). The finding in the expressions of TJPs showed that PCP-g down-regulated the expression of ZO-1 and Occludin, which subsequently enhanced permeability and facilitates the influx of water and electrolytes into the intestinal lumen, thereby contributing to the softening of feces. It indicated PCP-g was involved in the paracellular transport mechanism, which played a crucial role in the regulation of intestinal water absorption. The transcellular pathway functions via three specific molecular mechanisms: transmembrane flux facilitated by aquaporin water channels (AQPs), passive diffusion across the lipid bilayer, and solvent drag associated with active ion transport (48). The identification of AQPs has greatly enhanced our comprehension of the dynamics of epithelial water transport, especially regarding their essential contribution to the maintenance of intestinal water homeostasis (49, 50). AQPs, which are primarily found in the tissues of the digestive system, include 13 isoforms in mammals (9). Among these, AQP3 and AQP4 have been functionally confirmed to play essential roles in the regulation of water reabsorption in the colon (51, 52). Mechanistically, the G protein initiates the stimulation and activation of adenylate cyclase, which subsequently results in an increase in cyclic AMP (cAMP) concentrations. This elevation in cAMP levels facilitates the phosphorylation of protein kinase A (PKA). The activation of phosphorylated protein kinase A (P-PKA) plays a crucial role in regulating the expression levels of aquaporin 3 (AQP3), which in turn enhances water reabsorption (53–55). Our findings indicated that PCP-g interventions exacerbated this effect by inhibiting PKA phosphorylation, thereby influencing the expression of AQP3. Consequently, PCP-g produced laxative effects through two mechanisms: it disrupted tight junction integrity, as evidenced by a 41.7% and 40.7% reduction in ZO-1 and Occludin mRNA levels, and it downregulated AQP3 expression, resulting in an 83.4% decrease compared to the model group in membrane localization. Collectively, these alterations led to a 41.9% increase in fecal water retention (*p* < 0.01) when compared to the model group.

## 5 Conclusion

In this research, the polysaccharide PCP-g was isolated and characterized from *P. citrinopileatus*, and its therapeutic effects on sucralfate-induced constipation were examined. PCP-g was identified as a homogeneous polysaccharide with a molecular weight of 7.49 × 10^6^ Da, composed of Glc, Gal, Man, L-Fuc, Rha, GlcA, and Ara with a molar ratio of 1.00: 0.16: 0.13: 0.01: 0.006: 0.005: 0.006. The structural characterization of PCP-g revealed it to be a highly substituted β-(1→4)-glucan featuring a linear backbone that is periodically interrupted by side chains of 1,6-linked galactose and mannose, along with occasional branching at the 3,4 or 3,4,6 positions of the glucose units.

Furthermore, the oral administration of PCP-g alleviated sucralfate-induced constipation by increasing fecal water content, enhancing total fecal mass, and reducing the latency to the first pellet-like stool, in addition to enhancing intestinal motility. Additionally, PCP-g modulated the gut microbiota by reshaping microbial composition and promoting the production of SCFAs, while simultaneously decreasing gas production. Mechanistically, PCP-g downregulated the PKA-phosphorylated PKA-AQP3 signaling pathway, which resulted in a reduction of AQP3 overexpression and an improvement in colonic epithelial permeability within the transcellular transport pathway, while reduction the levels of tight junction proteins in the paracellular transport pathway.

The investigation elucidated the physicochemical properties of PCP-g and demonstrated its therapeutic effects against sucralfate-induced constipation, which would facilitate its application in the food and pharmaceutical fields.

## Data Availability

The original contributions presented in this study are included in this article/Supplementary material, further inquiries can be directed to the corresponding authors.
