# Supplementary material for: Therapeutic efficacy of a galactoglucan from Pleurotus citrinopileatus in constipation: modulation of aquaporin signaling and intestinal barrier
Source: Front Nutr. 2025 Jul 14;12:1635487. doi: 10.3389/fnut.2025.1635487 (PMC12301303; doi:10.3389/fnut.2025.1635487)
Supplement: Supplementary file 1 [file Data_Sheet_1.pdf]

Supplementary Material

Therapeutic Efficacy of a Galactoglucan from Pleurotus citrinopileatus in Constipation: Modulation of Aquaporin Signaling and Intestinal Barrier

Yi Gao[a,b,1], Lan Deng[a,c,1], Yuanyuan Chen[a,d], Peiyou Qin[a], Yuanyuan Zhao[a], Xiaoyan Zhao[a], Wei Liu[e], Dan Wang[a*], Shuang Zhao[a*]

[a] Institute of Agri-Food Processing and Nutrition, Beijing Academy of Agriculture and Forestry Sciences, Beijing 100097, China
[b] Department of Stomatology, Beijing Xicheng District Health Care Center for Mothers and Children, Beijing 100053, China
[c] College of Food Science and Bioengineering, Tianjin Agricultural University, Tianjin, 300392, China
[d] College of Life Science and Technology, Mudanjiang Normal University, Mudanjiang 157011, China
[e] Institute of Plant Protection and Microbiology, Zhejiang Academy of Agricultural Sciences, Hangzhou 310021, China

* Correspondence:
Yi Gao and Lan Deng contributed equally in this work.
Corresponding Author
wangdanjgs@163.com (D Wang)
zhaoshuang@baafs.net.cn (S Zhao)

SFig.1

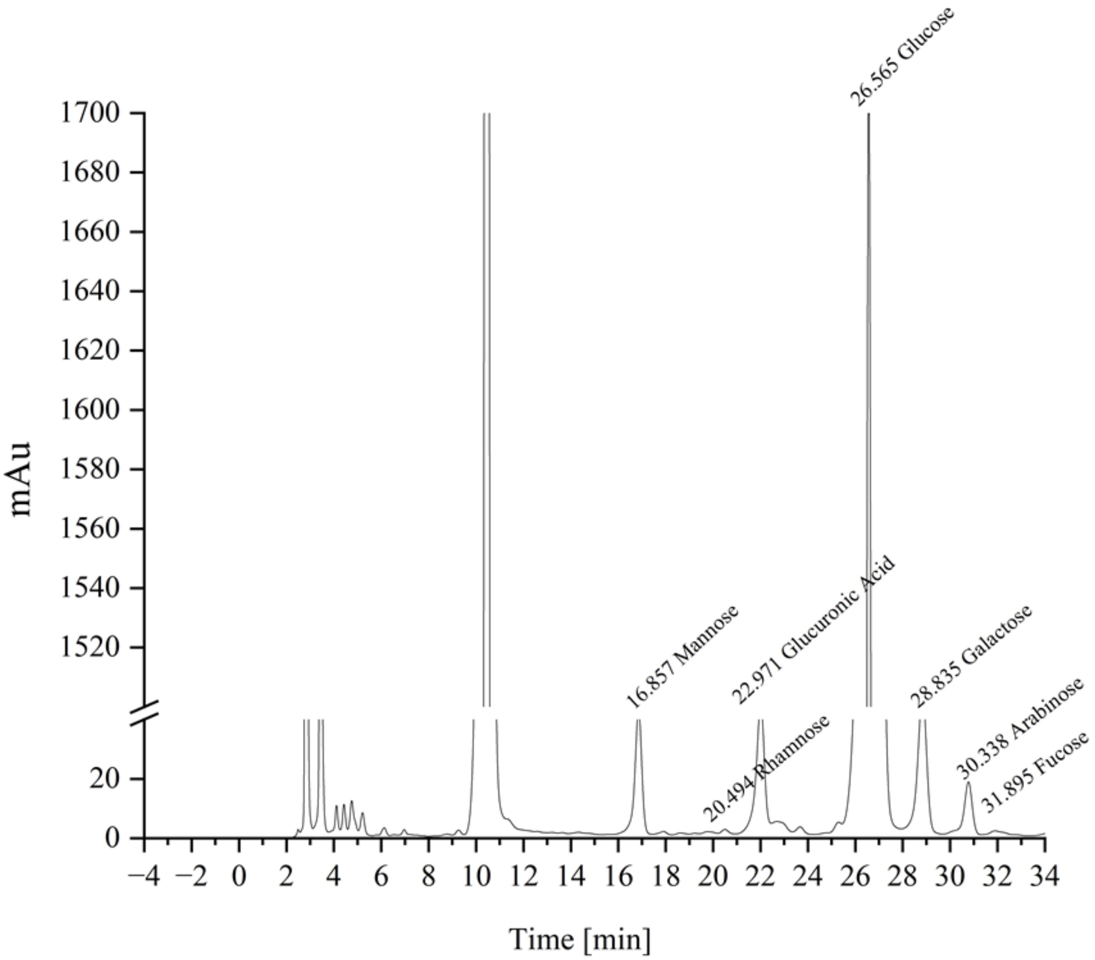

SFig. 1. The GC chromatogram of the standard monosaccharide mixture

SFig.2

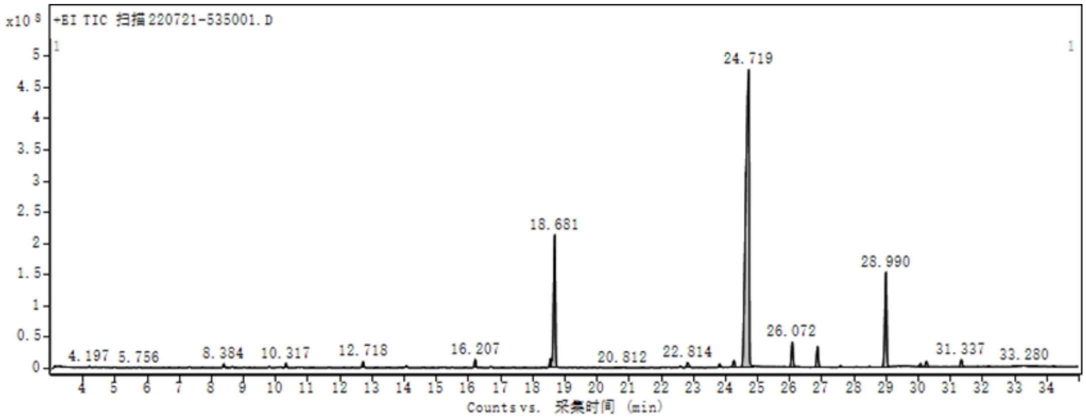

SFig.2 Total ion chromatography (TIC) by methylation analysis of PCP-g
